# Projected plastic waste loss scenarios between 2000 and 2030 into the largest freshwater-lake system in Southeast Asia

**DOI:** 10.1038/s41598-021-83064-9

**Published:** 2021-02-16

**Authors:** Alexander Matthew David Finnegan, Christos Gouramanis

**Affiliations:** 1 Geography Department, National University of Singapore, 1 Arts Link, #03-01 Block AS2, Singapore, 117570 Singapore; 2 Research School of Earth Sciences, The Australian National University, Building 142, Mills Road, Acton, ACT 2601 Australia

**Keywords:** Environmental impact, Sustainability, Environmental sciences, Limnology

## Abstract

Freshwater plastic pollution is critically understudied in Southeast Asia (SEA). Recent modelling studies indicate that SEA rivers contribute vast quantities of plastic to the world’s oceans, however, these fail to capture the complexity of individual systems. We determine the volume of mismanaged plastic waste (MPW) entering Tonle Sap Basin (TSB)—the largest freshwater lake–river system in SEA, between 2000 and 2030. Using economic, population and waste data at provincial and national levels, coupled with high resolution population and flood datasets, we estimate that *ca*. 221,700 tons of plastic entered between 2000 and 2020, and 282,300 ± 8700 tons will enter between 2021 and 2030. We demonstrate that policy interventions can reduce MPW up to 76% between 2021 and 2030. The most-stringent scenario would prevent 99% of annual MPW losses by 2030, despite substantially higher waste volumes and population. If successfully implemented, Cambodia will prevent significant losses in natural capital, material value and degradation in TSB worth at least US$4.8 billion, with additional benefits for the Mekong River and South China Sea.

## Introduction

Plastic pollution has emerged in recent decades as one of the greatest contemporary threats to global ecosystems, representing a major challenge for water quality, aquatic life and overall human wellbeing in the twenty-first century^1^. The ever-increasing global demand (and disposal) of plastics has led to profound changes to the natural world, as plastic has progressively leaked from the Anthroposphere^2,3^. Plastic has since evolved into one of the most-recent, novel, and widely-recognised pollutant in the environment^4^. Such is the comprehensive infiltration of plastic into the environment, across an entire spectrum of forms and size, plastic pollution is now irreversible and planetary-scale in nature^5^. Consequently, plastic has already met two of the three conditions of a planetary boundary threat^1,6,7^. It is unclear if or when plastic may exceed the final threshold of global systemic change to the planet^1,8,9^.

Plastic pollution in many aquatic systems remains critically understudied and most research has focused on the marine environment^10^. Freshwater is one of the most important natural resources available on Earth and the security of good-quality freshwater resources is an increasing global concern^11,12^. Plastic pollution is compounding the mounting contemporary issues of climate-change, eutrophication, invasive species, and existing, legacy damage, caused by nutrient loading, acidification and shoreline modification^12^. Specifically, the freshwater systems of Southeast Asia (SEA) are highly vulnerable, whilst the adaptive capacity of the population is comparatively low^13^. While studies of riverine plastic pollution in SEA have grown^14–17^, there are no dedicated systematic studies of plastic pollution in major SEA rivers, such as the Mekong River (MR)^10^.

Recent global modelling studies of riverine plastic pollution transported to the world’s oceans have demonstrated that SEA rivers contribute some of the highest quantities of plastic from continental interiors^18,19^. However, whilst these studies have been successful in producing large datasets, and identifying rivers or regions for further examination, the uniform approach used for global aquatic systems have failed to capture the complexities of individual systems. This would imply that a higher degree of accuracy in the global estimates of plastic losses could be achieved if key sites were examined in detail. To address this disparity between global-scale modelling, and local spatial- and temporal-scale complexity in assessing plastic pollution, we develop and apply a rigorous modelling framework to investigate the past, present and future volume of mismanaged plastic waste (MPW) that has entered Tonle Sap Basin (TSB: comprising Tonle Sap Lake (TSL) and Tonle Sap River (TSR)) in Cambodia. We target TSB as it is the largest freshwater lake–river system in SEA and of global significance due to the unique flood-pulse annual cycle, cultural and historical heritage^20^, and TSB’s connection with the MR, South China Sea and world’s oceans. In addition, TSB is a key site to directly investigate the impacts of MPW from a large population and a rapidly growing, lower-middle income country. Finally, we examine how targeted policy interventions could reduce MPW inputs into TSB and the growing global threat of MPW.

## Tonle Sap Basin

**Figure 1:**
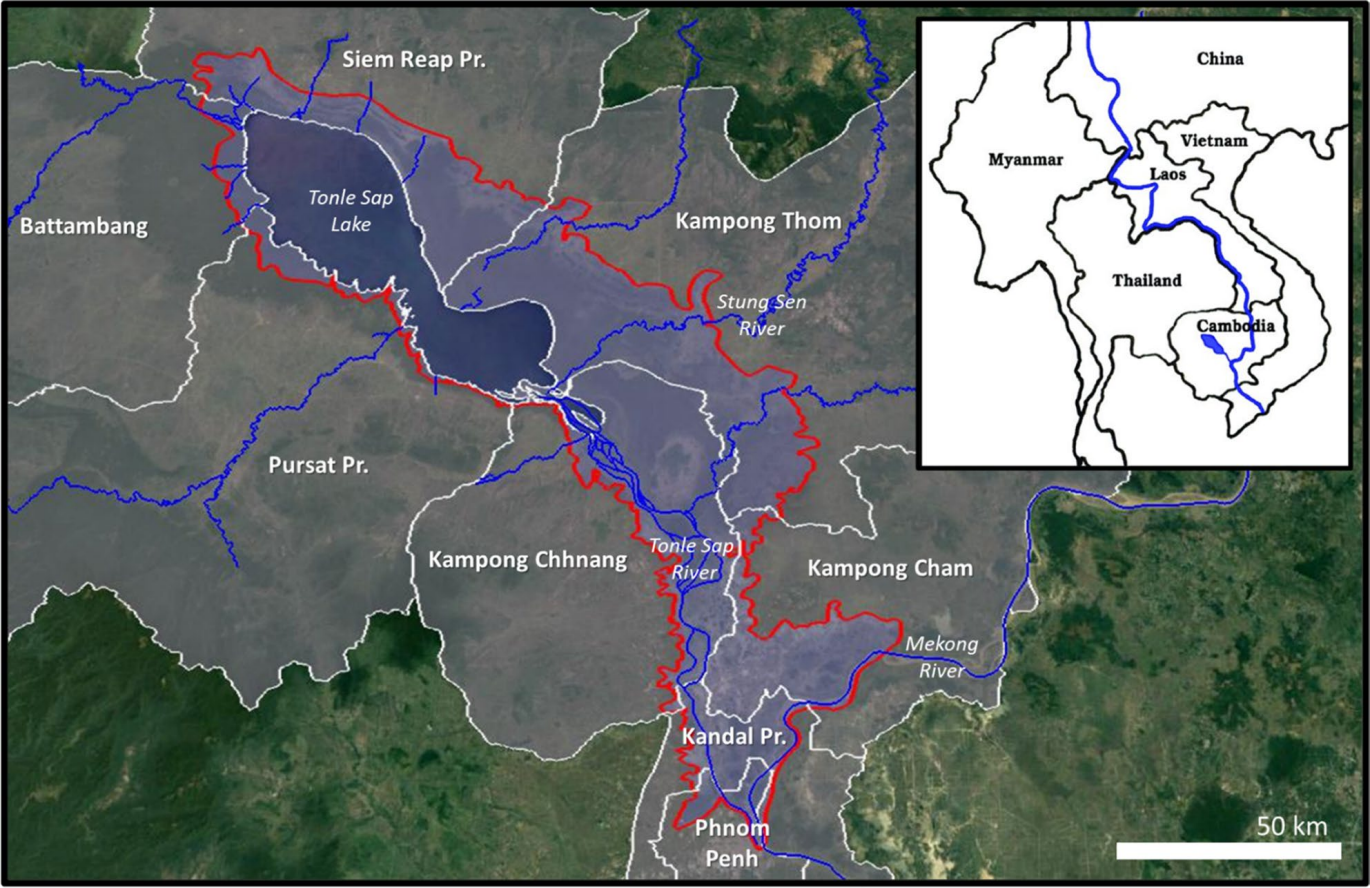
Map of study area and provinces around TSB. Red outline delineates the annual flood extent used in this study. Blue shaded area shows the extent of the perennial lake when floodwaters recede. Inset shows the location of the MR and TSB within SEA^60^. Map created in Google Earth Pro. Coordinates: 13°32′N 103° 8′E to 11°38′N 105°54′E. Eye alt: 305 feet. Landsat Copernicus. (2020).

TSB, situated within the 800,000 km^2^ MR Basin in SEA (Fig. 1)^21^, has a distinctive wet and dry season, causing water levels and hydrological inputs to the lake to fluctuate on an annual basis^22^. Every year, the southwest monsoon creates a flood-pulse upstream in the MR Basin. The pulse of water is subsequently propagated through the main stem of the MR towards TSL via the TSR at the confluence of rivers in Phnom Penh. The rapid increase in water level in the MR causes the TSR to reverse its flow towards TSL^23^.

TSB is the world’s fifth largest inland fishery and provides 50 to 80% of Cambodia’s animal protein^24^. Communities near and on the lake rely on the lake water for household purposes including drinking, cooking and cleaning with little to no treatment beforehand^25^. This poses a substantial risk for microplastic and associated contaminants to enter the local population. Plastic has many pathways into TSB, via the seasonal and dynamic hydrological changes, expansion of surface area from flooding, tributaries, wind and direct disposal into TSB. As the hydrological discharges leaving TSL via the TSR are significantly higher than those entering TSL^22^, this may suggest that TSB is a source of plastic for the MR.

## Results

### Mismanaged Plastic Waste losses projection from 2000 to 2030

**Figure 2:**
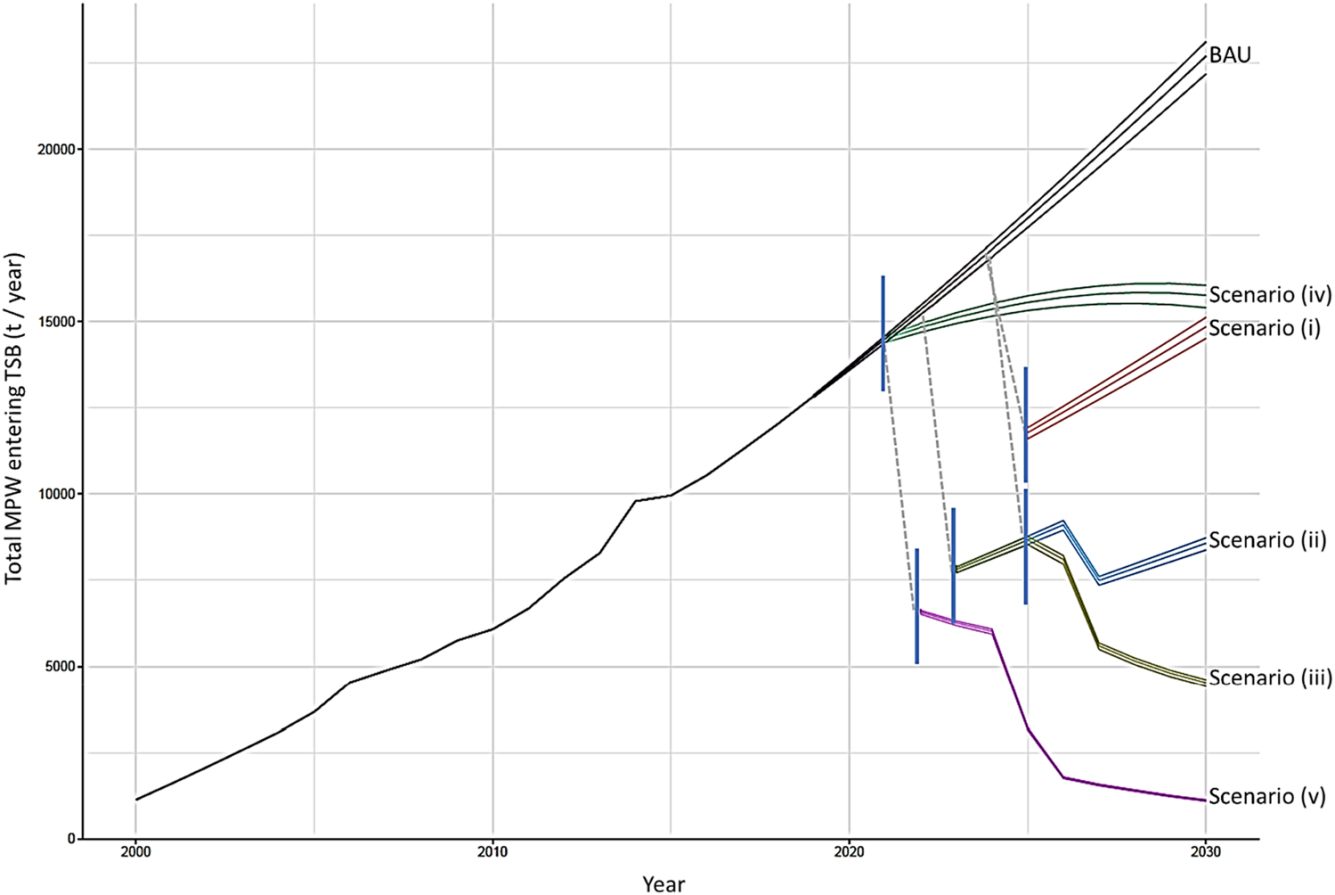
Five Scenarios (i to v) from 2021 to 2030, in addition to the Business as Usual (BAU) Scenario from 2000 to 2030. Error lines are shown as 95% prediction intervals.

We estimate that *ca*. 221,700 tons of plastic entered TSB between 2000 and 2020. In 2000 we estimate that 1,750 tons year^−1^ entered TSB, which rapidly increased between 19 and 41% per year until 2006 (Fig. 2). By 2010, we estimate that 9,411 tons year^−1^ entered TSB. The annual increase to 2020 is slower but still amounts to 21,062 tons year^−1^ entering TSB, representing a 124% increase from 2010. By 2030, under a Business As Usual (BAU) Scenario, we estimate that 34,392 tons year^−1^ will enter TSB equating to an additional *ca*. 282,300 ± 8700 tons between 2021 and 2030 (Fig. 2).

**Figure 3:**
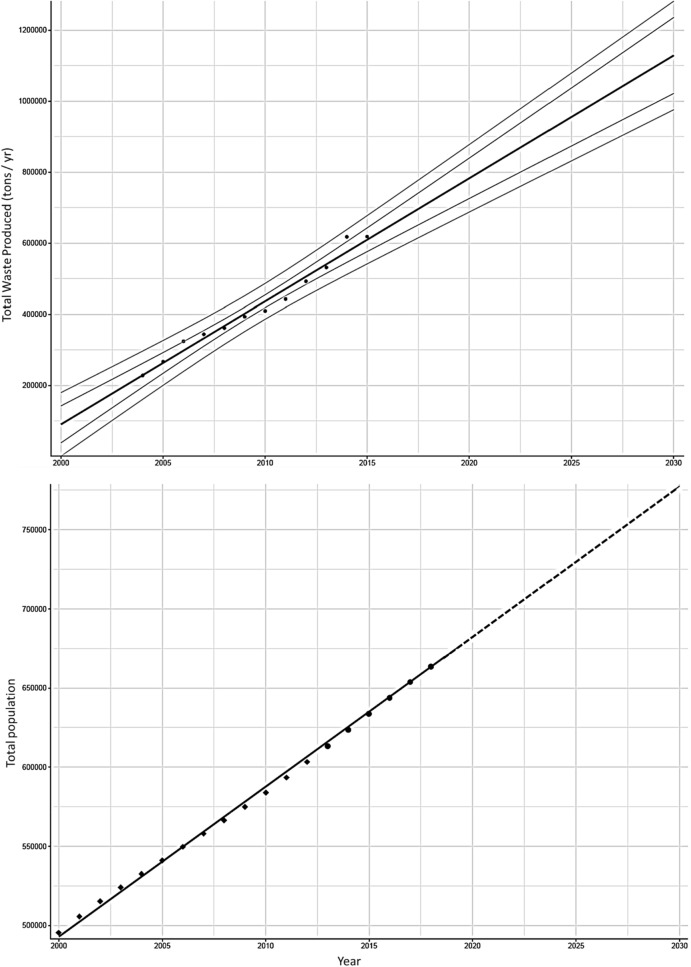
(top) Total waste disposed of in tons per year in Phnom Penh, between 2004 and 2015^28^. Data has been projected forwards and backwards between 2000 and 2030 (black line), with prediction and confidence interval at 95%. (bottom) Total Cambodian population from 2000 to 2018^54^. Total population is projected forward from 2019 to 2030, using the years 2013 to 2018 (dashed line).

The estimated ${\text{Annual}}\;{\text{ Average}}\;{\text{ Mismanaged }}\;{\text{Plastic }}\;{\text{Waste }}\;{\text{Generation}}\;{\text{ Rate }}({\text{MPWGR}}_{Avg} )$ increased from 1.8 kg (2000) to 8.2 kg (2010) and 15.7 kg (2020), and will increase to 22.6 kg (2030). Growth was pronounced in the 2000s, following the high annual growth rates in population and waste generation at the start of the decade (Fig. 3). In the 2020s, although the growth rate is expected to be much lower, in absolute terms, the weight increase will be similar to the 2000s and 2010s. At the provincial scale the change in ${\text{Annual }}\;{\text{MPWGR}}_{Prov}$ is profound with Siem Reap (highest initial value) growing from 3.88 kg (2000) to 48.7 kg (2030) and Pursat (lowest initial value) growing from 0.06 kg (2000) to 0.75 kg (2030).

### Policy intervention and impact on plastic inputs

Scenario (i) and its reduction of plastic bags by 50% results in a decrease of *ca*. 8,200 tons of plastic from entering TSB in 2025. By 2030, however, the growing population results in MPW increasing under this scenario. In this scenario, an additional 218,200 ± 6500 tons of plastic will enter TSB between 2021 and 2030. A total of *ca*. 64,000 tons of plastic is prevented from entering TSB under this scenario, compared to BAU (Fig. 2).

Scenario (ii) causes a large, immediate drop in plastic in 2022 of *ca*. 11,000 tons, with a second drop of *ca*. 2300 tons in 2026 from the previous year. After 2026 MPW again rises due to the growing population. Between Scenario (ii) and BAU, there is a reduction of *ca*. 21,000 tons of plastic entering TSB annually by 2030. In this scenario, an additional 131,100 ± 4200 tons of plastic will enter TSB between 2021 and 2030. A total of *ca*. 151,000 tons of plastic is prevented from entering TSB under this scenario, compared to BAU (Fig. 2).

Scenario (iii) results in MPW continuing to decrease after 2025, due to the continual improvements in recycling. These improvements see MPW from mixed recyclables reduce from *ca*. 6900 tons per year (2024) to *ca.* 2400 tons per year (2030), representing a total decrease of 66%. Between 2021 and 2030, implementation of Scenario (iii) will result in 105,400 ± 3500 tons entering TSB, which is *ca*. 177,000 tons less than BAU (Fig. 2).

Scenario (iv) results in a lower rate of MPW increase compared to the BAU Scenario from 2022, peaking at *ca*. 24,000 tons per year in 2028 before a slow decline until 2030. In this scenario, an additional 235,400 ± 7100 tons of plastic will enter TSB between 2021 and 2030. A total of *ca*. 47,000 tons of plastic is prevented from entering TSB under this scenario, compared to BAU (Fig. 2).

Scenario (v) causes a large initial annual drop by *ca*. 12,200 tons of plastic in 2022. The improved annual recycling ensures that MPW peaks in 2021 (*ca*. 12,300 tons) and decreases to 2030 (*ca*. 1200 tons). From 2025, foam plastic is eliminated from the waste stream and 80% collection coverage is implemented for all provinces (50% for Floating communities). In 2025 the combined policy improvements cause the second largest annual decline by (*ca*. 4300 tons). The last major decline occurs in 2026, with the plastic bag reduction of 90% initiating an annual drop of *ca*. 2200 tons. By 2030, the total annual MPW input into TSB is *ca*. 1700 tons, equating to 4.9% of the BAU Scenario. Between 2021 and 2030 approximately 66,800 ± 1700 tons is added to TSB, which is *ca*. 215,000 tons less than the BAU Scenario (Fig. 2).

## Discussion

The annual MPW losses into TSB from 2000 to 2018 follows the trend in Cambodian economic growth and development as the nation emerged from civil war, inclusion into the WTO and associated influx of foreign investment and goods to the present. Between 2000 and 2003, we predict the annual MPW input into TSB to increase 128% from 1750 to almost 4000 tons year^−1^. In 2004, Cambodia joined the WTO, which was a primary catalyst for growth, with gross domestic product (GDP) per capita increasing annually by *ca*. 16% between 2004 and 2008. Besides a minor contraction in 2009 coinciding with the global financial crisis, the GDP per capita has since increased by 6 to 12% annually^26^. The waste generation rates in this study are strongly correlated with GDP and population growth, which mirror global trends^27^.

Throughout the 2010s, the predicted MPW increases continuously except for 2015 (Fig. 3). In 2015, a significant slowing in total MPW growth was calculated based on an irregularity in the waste data from Phnom Penh, which increased by 16.2% between 2013 and 2014, and 0.1% between 2014 and 2015^28^. The cause of this irregularity is unclear but may be related to Phnom Penh’s municipal solid waste management being transferred to local government authorities in 2015, which faced initial difficulties^28^. The BAU Scenario predicts that waste continues to grow throughout the 2020s but gradually declines from a 6% annual increase in 2020 to 4% annual increase by 2030 (BAU, Fig. 2).

From 2021 to 2030, five scenarios are presented to explore how targeted polices could reduce MPW inputs into TSB (Fig. 2). This decade is crucial as by 2029, the amount of MPW entering TSB since 2021, will have exceeded the total input between 2000 and 2020. Decades under a BAU Scenario, would rapidly exacerbate the situation and pose a grim outlook for the future, with potentially more plastics in TSB than fish (by weight) as is predicted for the world’s oceans. For the world’s oceans, this crossover point is expected at *ca.* 2050^29^. Although a direct comparison cannot be made as TSB is an open-system and the total mass of fish has not been estimated, TSB produces approximately 500,000 tons of fish annually^24^. Whilst the total fish catch has remained stable over the past decade, this appears to be the ecological threshold for harvest levels, due to continuous evidence of fishery degradation and possible collapse for some species^30^. Under a BAU Scenario a cumulative 500,000 tons of plastic will have been lost into TSB by 2030.

**Figure 4:**
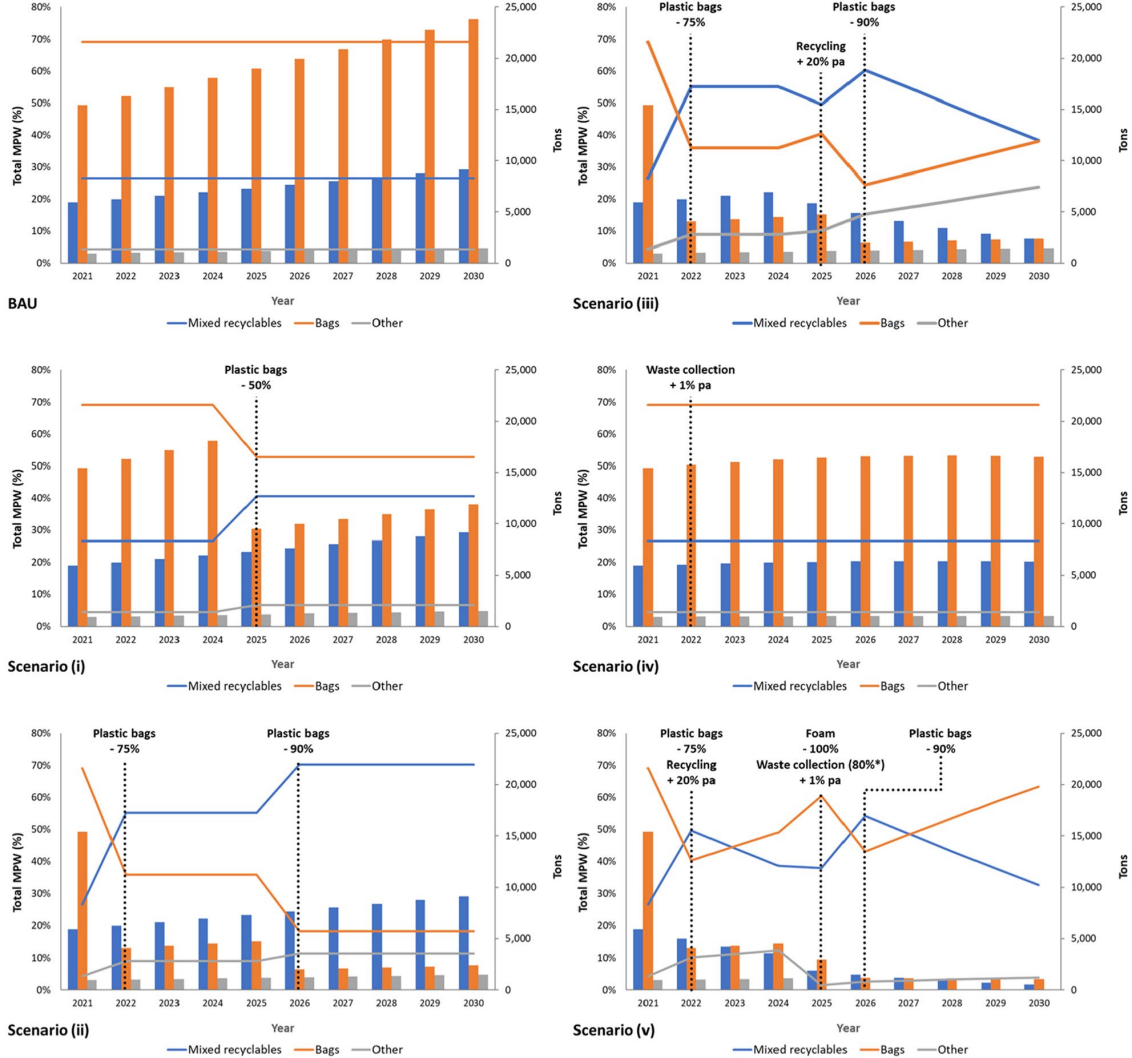
Analysis of Scenarios (i) to (v) and the impact that policy improvements have on individual components of the estimated MPW, which are: mixed recyclables (PET bottle, HDPE), bags and others (foam, film). Total annual change in MPW (tons) shown by bars and relative change (%) shown by lines. Note Scenario (v) *waste collection is 80% for all provinces in 2025, except for the floating communities with 50%.

In the scenarios presented, the biggest single decreases of MPW are observed with plastic bag reductions as plastic bags represent the largest plastic type (Table 1), based on commercial waste data from Phnom Penh^31^. However, due to ongoing population growth, plastic usage will inevitably increase. Scenarios (i) and (ii), which primarily target MPW from plastic bags, cuts cumulative MPW inputs into TSB by 23% and 54%, respectively (Fig. 4). The latter is significantly more aggressive and leads to major reductions in MPW, but ultimately, the population trajectory will subsequently increase MPW and highlights the need for more comprehensive measures targeting other plastics. Major reforms in Cambodia would be required to encourage the population to reduce plastic bag usage by 90% compared to current levels. Presently, Cambodia has a plastic bag charge, implemented in April 2018. The Royal Cambodian Government issued Sub-Decree 168 declaring the required thickness of plastic bags used in the country, and a fee of 400 Riel (US$0.10) for consumers, to be applied to each plastic bag used in all supermarkets and commercial centres. However, consumers are generally unaware of the country’s existing regulation on plastic bags, and there is a low rate of compliance from vendors or enforcement^32^. In addition, plastic bags in Cambodia have an array of applications beyond their initial usage in retail situations, including rain covers, storage-space, plant pots, waste bags and electrical insulation^33^. Therefore, a significant shift in consumer mindset would be required.

**Table 1:** Commercial solid waste generation in Phnom Penh^31^, used in this study to understand how policy changes may impact total plastic losses into TSB.

Plastic type	Composition of total plastic waste assumed in this study (%)	Policy improvement
PET bottle	8.3	Increased recycling
HDPE	18.2	Increased recycling
Film plastic	0.4	-
Plastic bag	69.1	Plastic bag reduction
Foam plastic	3.9	Ban on foam plastic
Total	99.9

Scenario (iii) builds upon Scenario (ii) but adds annual incremental recycling improvements that specifically target bottles and HDPE, thus reducing these potential sources of MPW (Table 1). These 20% annual improvements make a noticeable difference to plastic waste (Fig. 4). Following the plastic bag policy implementation in 2022, bags immediately reduce their relative abundance from 69 to 36% of the total, whilst mixed recyclables increase from 27 to 55%. This remains stable until 2025 and the recycling improvement implementation (Fig. 4). Such incremental changes in recycling rates seen in this scenario allow policies, infrastructure, and consumer behaviour to adapt and improve over time, bringing compounding improvements in future years. Whilst the policies to reduce plastic bags are effective at reducing the most abundant plastics, this source of plastic increases towards 2030 as the population grows. This highlights the issue of increasing population and the associated resource demands which are required. Scenario (iii) cuts cumulative MPW inputs into TSB by 63%.

Scenario (iv) results in the second highest MPW input into TSB (after BAU), with just a 17% reduction between 2021 and 2030 (Fig. 4). Scenario (iv) appears to be one of the least successful scenarios, however, the cumulative improvements to TWC would result in some of the fastest and most pronounced changes in the 2030s, if improvements were sustained. Under the BAU Scenario, the TWC averages 69%, which is akin to the average estimated collection rate in East Asia and Pacific (71%). By 2030, the average collection rate under Scenario (iv) sits at 78%, which would exceed the average estimate for urban areas in East Asia and Pacific^27^. This scenario shows that increasing collection coverage is a crucial tool in reducing MPW in TSB. However, in the short term, increasing collection coverage cannot bring about dramatic changes, unless more ambitious and immediate annual targets are met. One advantage to this strategy is that the only requirement is a compliance to waste collection services and does not require major changes to consumer habits.

Scenario (v) presents the most stringent policy implementation that targets all major sources of MPW through immediate reductions and incremental improvements to recycling, whilst setting a universal standard for waste collection coverage that is incrementally improved. Scenario (v) would be the most challenging to implement, navigating the benefits that plastic provide in public hygiene, productivity, and economics, whilst not further endangering the health of TSB and the surrounding environment. However, Scenario (v) would cause a remarkable improvement to annual MPW losses into TSB. By 2030, the amount of MPW would have a downwards trajectory, with the annual input reaching parity to the annual input in 2000 (Fig. 2). Scenario (v) cuts cumulative MPW inputs into TSB by 76%. It is unlikely that loss of plastic waste could ever be fully eliminated based on the steps outlined in these scenarios. Although, if losses could be reduced to around 5% each year, this would be a remarkable result and would reach parity with Europe and the US^29^. Based on a BAU Scenario, the total amount of plastic waste generated within the study area, assuming plastic represents 15.5% of total waste, would be approximately 243,000 tons in 2030. This would mean that both Scenario (iii) and (v) would be well within this 5% threshold. By 2030, Scenario (v) would prevent more than 99% of the plastic waste generated annually from entering TSB. Nevertheless, over 65,000 tons of MPW would still enter TSB between 2021 and 2030 under Scenario (v). This amount cannot be avoided unless policy changes were implemented more rapidly or to higher standard of regulation.

As MPW is typically not determined in most waste studies, we compare the WGR changes in TSB with those from other regions. World Bank per capita waste generation rate estimates for East Asia and the Pacific region published in 2012^34^, ranged from 0.4 to 4.3 kg capita^−1^ day^−1^, which are considerably higher than our estimated value between 0.5 and 0.6 kg capita^−1^ day^−1^ for TSB in 2012. Subsequent World Bank estimates published in 2018^27^, decrease their Cambodian estimated WGR values to 0.2 kg capita^−1^ day^−1^ in 2016, increasing to 0.25 kg capita^−1^ day^−1^ in 2030. Conversely, recent United Nations Environment Programme (UNEP) estimates suggest an average between 1.1 – 1.2 kg capita^−1^ day^−1^ for East Asia and Pacific from the mid-2020s^35^. In our study, we predict values of 0.77 kg capita^−1^ day^−1^ (2016), increasing to 0.94 kg capita^−1^ day^−1^ (2020) and 1.36 kg capita^−1^ day^−1^ (2030). Our estimates are comparable to the UNEP^35^ analyses but differ substantially from the World Bank’s estimates^27,34^. This disparity indicates the fragmented, inconsistent, and contradictory waste estimates in the public domain over the past decades, making such a comparison between studies problematic.

Our approach is analogous to the framework developed by Jambeck et al.^36^ (J15 hereon), but several important differences exist. In both studies, proportional increases in MPW have been calculated across relevant timeframes. J15 used population projections for 2015, 2020 and 2025, and calculated annual growth in plastic percentage of the waste stream, whilst keeping the waste generation rates per capita and inadequately managed waste percentages constant. Plastic was calculated through a conservative linear increase of 0.19% per year (SE = 0.06%). We adopted a different approach to calculate MPW focusing on a variable waste generation rate per capita by province, with constant values for plastic percentage and TWC. The fixed values for plastic percentage (between Low, Middle, and High) is more appropriate for TSB, given the vastly different spatial scales of the two studies and how the plastic percentage in the waste stream varies significantly geographically from reported figures in Cambodia^37^. We use a higher plastic percentage in the waste stream compared to J15. When comparing the MPW generated, J15 calculate a value of 21.3 kg capita^−1^ year^−1^ for the coastal zone of Cambodia in 2010, whereas we use a value of 8.2 kg capita^−1^ year^−1^.

We use a tiered approach in determining conversion rates, emphasizing the decreasing likelihood of plastic entering the aquatic system as distance increases from the annual flood extent, instead of providing a fixed high, medium, and low rate for the entire study area. A criticism of both studies is that the quantitative spatial/distance relationship for conversion rates has not yet been universally established. We have taken a more conservative approach by extending the sphere of influence an additional 5 km from the flood boundary compared to the 50 km used by J15. The boundary delineating the flooded area in our study does not typically exceed the permanent lake by > 20 km. Using J15′s conversion rates would yield between 5800 (15%) and 15,500 (40%) tons of plastic in TSB in 2018 (within 5 km of the flood boundary). To obtain the value of 18,655 tons per year in 2018 (middle-value) in our study, this would equal a conversion rate of 48%, meaning half of MPW produced annually enters TSB.

Two recent global-scale catchment-to-ocean models both used MPW as a key component to estimate macro- to micro- plastic fluxes in rivers^18,19^. Schmidt et al.^19^ (S17 hereon) estimate that the MR transports between 6300 and 37,000 tons of plastic to the ocean annually, of which, only 3300 tons are macroplastics. Similarly, S17 estimate that *ca*. 1,931,000 tons of MPW are generated annually in the MR catchment, meaning that less than 0.2% of the total MPW produced in the MR catchment enters the global oceans. Lebreton et al.^18^ (L17 hereon) estimate that the MR transports 22,800 tons of plastic annually, of which between 3900 and 7500 tons are estimated to be macroplastics. Our 2018 estimate of 18,655 tons (middle-value) entering only TSB suggests that both S17′s and L17′s estimates are too low. Due to the substantial amounts of MPW being generated in the Lower Mekong catchment, large flood system and lack of any anthropogenic or natural barriers to the South China Sea (SCS), the estimates from L17 and S17 should be treated with scepticism. However, neither study intended to scrutinise and detail individual watersheds.

The current loss of plastic into TSB poses several local and regional challenges. Firstly, the economic problem that plastic presents to Cambodia. To clean-up a large and complex system such as TSB would be non-viable, requiring immense resources which Cambodia would struggle to complete without international financial and technical assistance^12^. Under this assumption, the *ca*. 221,700 tons of plastic which has already entered TSB will remain, or, will be gradually removed through the annual flooding and emptying of the lake. In the coming years, however, a lucrative opportunity could be cultivated by preventing plastic from entering TSB. Using the prices of recycled plastic in the EU as a benchmark, which fluctuated between 230 and 370 €/ton from 2004 to 2018^38^, would mean Cambodia was currently losing €4,300,000 to €6,900,000 per year into TSB. Under a BAU Scenario of 282,300 ± 8700 tons of plastic lost between 2021 and 2030, this would equate to €65,000,000 to €104,000,000 worth of plastic. Nevertheless, plastic pollution is not just a loss of material value. Recently, the true cost of marine plastic was placed at between US$3,300 and US$33,000 per ton of plastic every year, with quantifiable costs to fisheries, aquaculture, recreation, heritage and health of ecosystems^39^. While this cost was placed on marine plastic, many similar interactions will still be occurring in a large freshwater system like TSB, including entanglements, ingestion, and transport of pollutants, affecting all trophic levels. Excluding the loss in material value, the cumulative costs in natural capital between 2021 and 2030 would be US$4.8 billion using the lower-end estimate. Owing to the transboundary nature of TSB and the MR, the financial liability of plastics entering Vietnam, and ultimately the SCS, may need to be considered in the future.

Unsurprisingly, plastic is not the single pressure that TSB is currently facing. Like many other large lake systems around the world, TSL is under severe threat from several compounding factors^12,13,40^. Aside from climate change and the challenges of dam building on the MR, issues such as population growth, increasing land-use intensity, and the general mismanagement of terrestrial and aquatic resources need to be addressed immediately^20,41^. Recent deforestation rates in the unique flooded-forest ecosystem around TSL are thought to be some of the highest, globally^42,43^. A burgeoning population has increased pollution from various sources including household sewage and agricultural run-off, which includes sediment, pesticides, and fertilisers^20,44^. Years of overfishing has led to a decline in slower-growing, higher-trophic species, whilst smaller-bodied, faster-growing, low-trophic fish have increased^30,42^. Plastic pollution is a relatively novel pollutant to TSB, yet it is aiding the degradation of this aquatic system with other recent and longer-term stressors. In singular terms each threat may be managed accordingly, although together, they show disturbing evidence of collective harm, acceleration of pollution and a legacy of ecological and economic costs for future generations^12^.

## Methods

### Data sources

As TSB undergoes profound spatial and temporal changes during the flood-pulse cycle we digitised a conservative boundary using a 30 × 30 m pixel resolution, estimated flood extent raster dataset, which covered the period from April 2013 to December 2015 in the Lower Mekong Basin^45^. The maximum extent of the lake during this period (i.e. 2013 to 2015) reached an estimated 14,795 km^2^ during the flood of 2013, however, this was a relatively exceptional year as the average estimated flood extent was 12,001 km^2^ between 2000 and 2014^46,47^. The total flood extent used in this study, following a conservative approach when digitising the April 2013 to December 2015 estimated flood extent, was 10,392 km^2^—i.e. 13% smaller than the estimated average between 2000 and 2014 (Fig. 1). This was due to omitting major TSL tributaries such as the Stung Sen in the boundary, which expand significantly during the wet season^48^.

We have assumed that the boundary delineating the flooded area is representative for the time period examined between 2000 and 2030, although the maximum lake extent naturally fluctuates interannually. Between 2000 and 2014, the estimated maximum flood extent oscillated between 8492 km^2^ in 2003 and 16,508 km^2^ in 2011. The flood extent used in this study would be the 11th largest (out of 15) between 2000 and 2015^46^. The flooded area intersects 8 provinces (Battambang, Kampong Cham, Kampong Chhnang, Kandal Province, Kampong Thom, Phnom Penh, Pursat Province and Siem Reap Province; Fig. 1). To create the flood boundary, we include all overland-flow north of the MR as this mass of water is typically included in water balance assessments of TSL^22^.

The provincial waste production data, which includes the waste generation rate per capita (0.03 to 1.38 kg day^−1^), and the amount of waste collected (%) for 2015 at the provincial level, comes from a recent government report^49^. The average waste generation rate was independently validated by Creaser et al.^50^ who estimated that Cambodian rural areas produced 0.4 to 1 kg day^−1^ per capita. Data for Kampong Cham and the Floating communities was not available, therefore data from Phenom Penh and Pursat Province substituted, respectively. For the Floating communities we used a Total Waste Collection (TWC; %) percentage of 0. We assume that collected waste is not mismanaged and does not enter TSB.

We examine a range of values for the percentage of plastic in the waste stream, a high-value of 19.32% based on the commercial waste generation in Phnom Penh^51^, a middle-value of 15.5% based on municipal solid waste management data in Phnom Penh^28^, and a low value of 10% from a Cambodia-wide estimate of municipal solid waste management^34^. We assume that the middle-value to be the likely average for TSB, based on the predominantly urban/semi-urban population.

We obtained an estimate of Cambodia’s population and population density from Facebook Connectivity Lab (FCL) and Center for International Earth Science Information Network Columbia University (CIESIN)^52^. The dataset estimates human population distribution at a resolution of 1 arc-second (approximately 30 m), providing highly accurate estimates of population and density in 2018. Provincial boundaries were obtained from United Nations Office for the Coordination of Humanitarian Affairs Regional Office Asia and Pacific^53^. The provincial boundaries exclude the lake, where many floating communities exist. We added 7236 people living in floating communities based on the FCL and CIESIN data^52^. Using ArcGIS Pro, the population data in GeoTIFF format was firstly converted to points, then clipped to the outline of the flooded area. Subsequently, the population of the flooded area was clipped to provincial level, to establish the total population for each province in the flooded area. Additional buffer zones of 1, 2, 3 and 5 km were created around the total extent of the flooded area, and the total population for these zones was established. The Summary Statistics function in ArcGIS was used to extract the population of each province and within each buffer zone.

### Numerical model creation

Using the definition by J15, MPW is defined as plastic material that is either littered or inadequately disposed. In this study we assume no littering as there is a blurred line between littering and disposing of domestic solid waste in Cambodia. Instead, we base the amount of MPW on the TWC percentage at provincial level (Table 2). We have estimated the amount of MPW that is produced on a per-capita basis in each province and multiplied this by the amount of people living both within the province and in the annual flood extent (Table 2).

**Table 2:** Annual mismanaged plastic waste generation rate ($\mathrm{Annual }{\mathrm{MPWGR}}_{Prov}$) per capita and total mismanaged plastic waste $\mathrm{Annual} {\mathrm{MPW}}_{Tot}$ in 2018 within the flood boundary.

Province	Flooded Population	WGR capita^−1^ day^−1^ (kg)	TWC (%)	Plastic (%)					
				Middle (15.5)		High (19.32)		Low (10)	
				MPWGR_Prov_ (kg)	MPW_Tot_ (tons)	MPWGR_Prov_ (kg)	MPW_Tot_ (tons)	MPWGR_Prov_ (kg)	MPW_Tot_ (tons)
Battambang	4311	0.63	86	5.07	22	6.32	27	3.27	14
Kampong Cham	130,085	1	83	9.43	1227	11.76	1529	6.08	792
Kampong Chhnang	124,730	0.4	62	8.66	1080	10.79	1346	5.58	697
Kandal Province	146,340	0.83	54	21.54	3152	26.85	3929	13.90	2034
Kampong Phom	35,495	1	52	27.27	968	33.99	1206	17.59	624
Phnom Penh	161,931	1	83	9.43	1527	11.76	1904	6.08	985
Pursat Province	5356	0.03	72	0.47	3	0.59	3	0.30	2
Siem Reap Province	48,170	1.38	61	30.78	1483	38.36	1848	19.86	956
Floating communities	7236	0.03	0	1.70	12	2.12	15	1.10	8
Total	663,656				9473		11,807		6112

The annual Mismanaged Plastic Waste Generation rate for each province (${\text{Annual }}\;{\text{MPWGR}}_{Prov}$) in kg capita^−1^ is calculated using:

1 $${\text{Annual}}\;{\text{MPWGR}}_{Prov} { } = \left( {\left( {WGR*365} \right)*P} \right)*\left( {1 - TWC} \right),$$

where WGR is the Waste Generation Rate per capita in the province, P is Plastic (%) in waste stream (constant values between middle, high, and low) and TWC is the Total Waste Collected in each province.

From Eq. (1), we then calculate the total annual Mismanaged Plastic Waste (${\text{Annual}}\; {\text{MPW}}_{Tot}$) in kg produced in each province:

2 $${\text{Annual}}\;{\text{MPW}}_{Tot} = Pop_{Prov} *{\text{Annual }}\;{\text{MPWGR}}_{Prov} ,$$

where $Pop_{Prov}$ is the provincial population within the annual flood extent.

The average annual Mismanaged Plastic Waste Generation Rate (${\text{Annual }}\;{\text{MPWGR}}_{Avg}$) in kg capita^−1^ for the total flooded area in all provinces, is calculated using the following equation:

3 $${\text{Annual }}\;{\text{MPWGR}}_{Avg} = \frac{{Total\; {\text{Annual}}\; {\text{MPW}}_{Tot} }}{Total \;Flooded\; Population}$$

In this study we have used conversion rates to estimate the amount of MPW which successfully enters TSB at the maximum flood extent (Fig. 1). A tiered approach is taken, with the sphere of influence extending an additional 5 km outwards from the annual flood extent boundary. Using buffers of 1, 2, 3 and 5 km, the total unique population was calculated for each individual buffer (Table 3). Adapting Eq. (2), the total MPW was determined for each buffer, using the total population of the buffer and the ${\text{Annual}}\;{\text{MPWGR}}_{Avg}$. The total MPW for each buffer was then multiplied with the conversion rate for that buffer. Here, we have assumed that the likelihood of MPW being captured by floodwaters decreases in distance away from the lake. Beyond 5 km, we have assumed that MPW will not be captured. We assumed that MPW within the flooded area would achieve a 100% conversion rate. The conversion rates for 1 (60%), 2 (40%), 3 (25%) and 5 (15%) km from the lake were based on the figures used in J15. Unlike J15, we extend the sphere of influence 5 km from the shoreline around TSB and have a tiered conversion rate that more accurately depicts the local physiography and demography.

**Table 3:** Total estimated plastic input into Tonle Sap Basin in 2018.

Conversion Rate	Buffer	Population	Middle (15.5%)	High (19.32%)	Low (10%)
100	0 km buffer	663,656	9473	11,807	6112
60	1 km buffer	435,941	3734	4654	2409
40	2 km buffer	383,365	2189	2728	1412
25	3 km buffer	425,264	1518	1892	979
15	5 km buffer	813,655	1742	2171	1124
	Total	2,721,880	18,655	23,252	12,035

### Population and (plastic) waste projections

To provide a cumulative estimate of mismanaged plastic waste entering TSB and how policy interventions could subsequently reduce these estimates, a 30-year numerical model was created for the area within the annual flood extent. From 2018, the population and MPW were projected forwards to 2030 and backwards to 2000. Anecdotally, plastic products have probably been in Cambodia since *ca*. 1994 CE following the end to hostilities with the Khmer Rouge^32^. However, the starting year of 2000 was chosen as Cambodia joined the Association of Southeast Asian Nations (ASEAN) in 1999, which marked the major transition of the country opening its borders to foreign goods and investment. We assume that this is the start of when plastic goods became widespread in the country, and particularly after 2004, when Cambodia joined the World Trade Organization (WTO).

We determined provincial and buffer zone populations to 2030 using national population growth trends between 2000 to 2018^54^ and assumed a linear, annual increase to 2030 (Fig. 3). Figures for both the total population and the percentage population growth rate in Cambodia, were obtained from World Bank estimates. The calculated projected annual population percentage growth rate decreases from 1.46 in 2019 to 1.10 in 2030 (R^2^ = 0.98).

Long-term, accurate and publicly available waste data in Cambodia is sparse, except for several major cities. Here, data were obtained for the total annual municipal solid waste collected in Phnom Penh and disposed of at a municipal dump site, between 2004 and 2015 in tons year^−1^^28^. The data, which showed sustained annual growth in total waste, were assumed to be representative of the population living across all provinces in the annual flood extent. The actual and calculated annual percentage waste increase were used to project forwards and backwards the $WGR$ (Eq. 1). The total waste collected and disposed of in Phnom Penh in 2004 CE was 228,000 tons year^−1^, rising to 618,000 tons year^−1^ in 2015, representing an increase of 171%, or, approximately 14% per year^28^. Spoann et al*.*^28^ estimated that *ca*. 20% of the total waste generated in Phenom Penh was not collected, which is similar to the figure of 83% TWC used in this study for Phnom Penh (Table 2). Total waste in Phnom Penh was linearly projected forwards from 2016 to 2030 and backwards from 2003 to 2000 (R^2^ = 0.97; Fig. 3). With the projected total annual amount of municipal solid waste collected in Phnom Penh, we then calculated the percentage annual growth rate for each year.

The plastic waste for each province and year were projected forwards and backwards from 2018, calculating the ${\text{Annual}}\;{\text{MPWGR}}_{Prov}$ with $P$ and $TWC$ constant from 2000 to 2030 (Eq. 1), the ${\text{Annual}}\; {\text{MPW}}_{Tot}$ (Eq. 2) and the ${\text{Annual }}\;{\text{MPWGR}}_{Avg}$ across the annual flood extent (Eq. 3). The ${\text{Annual}}\;{\text{MPWGR}}_{Avg}$ for all provinces was multiplied by the total population in each buffer zone (0, 1, 2, 3 and 5 km) for each year, and the respective conversion rates (100%, 60%, 40%, 25% and 15%) for each buffer zone was then applied. The summed totals equate to the total MPW estimated to enter TSB for each year from 2000 to 2030.

### Targeted policy scenarios

The scenarios presented in this study explore what impact policies would have on projected MPW, from 2021 to 2030. Two broad approaches were explored, the first was a reduction in certain plastic types becoming waste. The second was improvement in collection coverage across the annual flood extent. For the former approach, data from commercial solid waste generation in Phnom Penh^31^, which detailed the weight of plastic generated by type, was applied to the total MPW values projected for each year (from 2021 to 2030), province and buffer zone (Table 1). From this, policy improvements were proposed which included plastic bag reductions, a ban on foam plastic and increased recycling rates (which was applied to PET Bottle and HDPE plastic types only, i.e. mixed recyclables). For the second approach, incremental annual improvements to $TWC$ (%) was applied to each province.

Purposely, we do not propose how these scenarios could be implemented but assume a positive outcome of policies to reduce certain plastic types in the waste stream or improve waste collection. Successful policies typically use market-based instruments (MBIs) to reach a desired outcome. MBIs may include taxes, charges, fees, fines, and penalties, under the premise that either the polluter and/or user pays, and provides a full cost for recovery of the item^55^. The scenarios have been developed to be representative of model case-studies in reducing plastic waste around the world. Such successes include; reductions of plastic bag usage between 50 and 90% within a year^56–58^, plastic bottle recycling rates which have achieved rates of > 90%^29,59^ and a pilot study in Cambodia, extending waste coverage to a rural community which achieved a swift compliance rate of > 90%^37^. The scenarios we model are:

(i) Plastic bag reduction of 50% from 2025*
(ii) Plastic bag reduction of 75% between 2022*/90% from 2026*
(iii) Plastic bag reduction of 75% between 2022 to 2025*/90% from 2026*/Recycling increase (20% pa from 2025)
(iv) From 2022 a + 1% annual increase in waste collection to 2030
(v) Plastic bag reduction of 75% from 2022*/90% from 2026*/Recycling increase (20% pa from 2022)/Foam plastic eliminated from 2025/From 2025 at least 80% collection coverage (+ 1% annual improvement thereafter) and 50% coverage in floating communities

*reduction calculated from pre-intervention period.
